# Sources of reproductive health information and their associations with self-reported contraceptive familiarity and education needs among female college students: a cross-sectional study in China

**DOI:** 10.3389/fpubh.2026.1949119

**Published:** 2026-09-18

**Authors:** Yan Wang, Ting Feng, Danning Wang, Yan Ke, Meihua Wu, Lin Shen, Mei Wang, Kuimei Zhang, Ya Liu, Xiaoxia Wang, Wenming Cao, Xingdi Qiu

**Affiliations:** 1 Department of Gynecology, Shenzhen Hospital of Integrated Traditional Chinese and Western Medicine, Shenzhen, China; 2 Department of Gynecology, Pingshan District Central Hospital of Shenzhen, Shenzhen, Guangdong, China; 3 Department of Gynecology, Shandong Changle People's Hospital, Weifang, Shandong, China; 4 Department of Gynecology, Shandong First Medical University Affiliated Heze Hospital (Heze Municipal Hospital), Heze, Shandong, China; 5 Department of Obstetrics and Gynecology, Shenzhen University General Hospital, Shenzhen, Guangdong, China

**Keywords:** contraceptive familiarity, female college students, health education, information source, reproductive health, school education

## Abstract

**Purpose:**

Female college students (FCSs) obtain reproductive health information from multiple sources. Although the Internet has become a major channel for health information seeking, the relationship between information source, contraceptive familiarity, and education needs remains unclear. This study examined primary information sources and their associations with self-reported contraceptive familiarity and education needs in a multi-province sample.

**Methods:**

A cross-sectional survey was conducted among FCSs from 24 universities across 12 provincial-level regions in China from January 2025 to May 2026. The primary exposure was the main information source, categorized as school education, Internet, popular science books, family education, peers/friends, and radio/television. Outcomes included higher contraceptive familiarity, need for education, perceived necessity of school-based courses, and desired topics. Multivariable logistic regression with clustered robust standard errors was used, with school education as the reference.

**Results:**

The Internet was the most common source (27.29%), followed by school education (21.47%) and popular science books (18.08%). School education had the highest proportions of higher contraceptive familiarity (69.71%), education need (56.67%), and course necessity (79.71%). Compared with school education, family education (aOR = 0.744, 95% CI: 0.621–0.892), peers/friends (aOR = 0.734, 95% CI: 0.608–0.885), and radio/television (aOR = 0.733, 95% CI: 0.582–0.922) were associated with lower odds of higher familiarity. The Internet (aOR = 0.951, 95% CI: 0.811–1.114) and popular science books (aOR = 1.042, 95% CI: 0.875–1.240) did not differ significantly from school education. However, effect sizes were modest (Cramer's *V* = 0.071–0.120) and Nagelkerke *R*^2^ values were low (0.057–0.105), indicating limited explanatory power. The most desired topics were STI/gynecological disease prevention (74.19%), healthy sexual behavior (70.91%), and contraception (69.14%).

**Conclusion:**

In this multi-province sample, the Internet was the leading information source, but school education was associated with the most favorable profile of contraceptive familiarity and education demand. Given the modest effect sizes and cross-sectional design, these findings should be interpreted as associations requiring further investigation rather than as evidence for strong causal effects. Universities should strengthen structured curricula, integrate reliable digital resources, and support students relying on informal sources. Due to the cross-sectional design, causal inferences cannot be drawn.

## 1 Introduction

Reproductive health education is an important component of health promotion for young women. During the transition from adolescence to adulthood, female college students (FCSs) may encounter questions related to menstruation, contraception, sexual behavior, prevention of sexually transmitted infections, gynecological symptoms, and reproductive health services (1). Adequate reproductive health knowledge can support informed decision-making, reduce avoidable reproductive health risks, and improve students' ability to seek timely and appropriate care (1, 2).

In recent years, the Internet has become a major source of health information for college students (3). Online platforms provide convenient access to information on contraception, sexual health, gynecological disease prevention, and health services. For sensitive topics such as reproductive health, digital sources may be especially attractive because they offer privacy and easy access (4). However, online information varies in quality, and students may encounter fragmented, incomplete, commercial, or inaccurate content (5).

School education is another important source of reproductive health information (6). Compared with informal or unstructured sources, school-based education has the potential to provide systematic, age-appropriate, and scientifically accurate information (7). It can also offer opportunities for clarification, guided discussion, and referral to health services. However, reproductive health education in university settings may vary in coverage, content, frequency, and teaching methods (1).

Family education and peer communication also play roles in reproductive health learning (8). Family members may provide early guidance and values related to sexual and reproductive health, but communication on contraception and sexual behavior may be limited by cultural norms, embarrassment, or insufficient parental knowledge (9). Peers and friends may be important sources of emotional support and shared experience, yet peer-provided information may be inaccurate or incomplete (10).

Existing studies on reproductive health among FCSs have examined various aspects, including reproductive health literacy (11, 12), school-based health education participation (13, 14), sexual and reproductive health communication (15, 16) and attitudes, and health service needs and access (17, 18). However, few studies have systematically examined information source as the primary exposure variable in relation to contraceptive familiarity and education needs. Studies describing health information sources among young people have primarily focused on source prevalence rather than comparing how different sources, such as school education, the Internet, popular science books, family education, peers/friends, and media, are differentially associated with knowledge and educational demand (19–22). This distinction is important because the same level of information exposure may have different implications depending on whether the information comes from school education, the Internet, family, peers, or media (6, 17, 23, 24).

The present study used data from a cross-sectional survey of 6,428 FCSs in China. The study focused on the primary source of reproductive health information and examined its association with self-reported contraceptive familiarity, perceived need for reproductive health education, perceived necessity of school-based reproductive health courses, and preferred education topics.

## 2 Materials and methods

### 2.1 Study design and participants

This cross-sectional survey was conducted among FCSs in 12 provincial-level administrative regions of China from January 2025 to May 2026. The 12 regions were selected to represent China's major geographic and economic areas: eastern (Shandong, Jiangsu, Zhejiang), southern (Guangdong, Hainan), central (Shanxi, Anhui, Hebei), western (Sichuan), northeastern (Heilongjiang), and the autonomous regions (Xinjiang Uygur Autonomous Region and Tibet Autonomous Region). This coverage was intended to capture variation in socioeconomic development, healthcare access, and cultural contexts that may influence reproductive health information sources and education needs. Eligibility criteria were consistent across all institution types: all participants were enrolled female students (junior college, undergraduate, or postgraduate) at their respective institutions, with no additional restrictions. Convenience sampling was used to recruit students from 24 universities, including nine comprehensive universities, eight medical universities or colleges, and seven vocational or technical colleges. These institution types represent the major categories of higher education institutions in China and were included to capture variation in students' educational backgrounds and potential exposure to reproductive health education. The institutions were selected based on geographic diversity and institutional accessibility rather than probabilistic sampling. In the primary analysis, participants from all institution types were pooled into a single analytical sample because: the research question about reproductive health information sources applies to all FCSs regardless of institution type; academic major (medical/health-related vs. non-medical) and educational level, which capture key dimensions of institutional differences, were included as covariates; and university-level clustering was accounted for using cluster-robust standard errors.

### 2.2 Data collection and quality control

Students were recruited through campus networks, class group messages, and social media platforms. The survey was administered via an online questionnaire platform (*Wenjuanxing*), and all items were set as required to prevent missing responses. Completion time was recorded automatically by the platform. A total of 6,608 questionnaires were collected. Data quality screening was performed using two criteria: logically inconsistent responses (e.g., contradictory answers to mutually exclusive items); and excessively short completion times (<120 s, approximately one-third of the median completion time of 360 s). Based on these criteria, 180 questionnaires were excluded, yielding 6,428 valid questionnaires (response validity rate: 97.28%). A participant flow diagram is provided in Supplementary Figure S2. Students from junior college, undergraduate, and postgraduate programs were included. The present analysis used variables related to reproductive health information source, self-reported contraceptive familiarity, reproductive health education needs, and relevant covariates. The full questionnaire items (Chinese and English) are provided in Supplementary File S1.

### 2.3 Measures

The questionnaire was developed based on a literature review and existing validated instruments. Content validity was assessed by three experts in reproductive health and public health. Face validity was evaluated through pilot testing with 50 FCSs, who provided feedback on item clarity and comprehensibility.

#### 2.3.1 Primary exposure: source of reproductive health information

The main exposure variable was the participant's primary source of reproductive health information, assessed by the question: “What is the main channel through which you currently understand reproductive health knowledge?” Response options were: popular science books (including printed books and electronic popular science articles), Internet, peers or friends, radio or television, family education, and school education. These six categories were selected based on: a review of the literature on health information sources among young people (23, 25, 26); the major channels through which reproductive health information is typically disseminated in China; and pilot testing of the questionnaire, which confirmed these categories captured the main sources reported (23, 27). School education was used as the reference category in regression analysis. School education was chosen as the reference category because it represents the most structured and potentially standardized form of reproductive health information delivery, against which other sources can be meaningfully compared (6, 27).

#### 2.3.2 Outcome: self-reported contraceptive familiarity

Self-reported contraceptive familiarity was assessed by asking participants: “How familiar are you with contraceptive methods such as the calendar/rhythm method, withdrawal, barrier methods (e.g., condoms), and medications (e.g., oral contraceptives)?” Response options were “very familiar,” “generally familiar,” “not very familiar,” and “no knowledge.” This measure reflects self-perceived familiarity rather than objectively assessed knowledge of contraceptive efficacy, correct usage, or side-effect profiles. The single-item approach may not capture method-specific knowledge differences (e.g., familiarity with condoms vs. oral contraceptives) and may be subject to social desirability and self-perception biases (28, 29). For logistic regression, “very familiar” and “generally familiar” were classified as higher self-reported contraceptive familiarity, while the other two categories were classified as lower self-reported contraceptive familiarity. Dichotomization was chosen to simplify clinical interpretation and because the distribution of responses was skewed (approximately two-thirds in higher familiarity categories). We conducted ordinal and multinomial logistic regression sensitivity analyses with the full four-category outcome, which yielded broadly consistent results (Supplementary Table S5).

#### 2.3.3 Outcomes: education needs and attitudes

Need for reproductive health education was assessed by asking: “Do you think you need reproductive health education?” Response options were “need,” “indifferent,” and “do not need.” For analysis, “need” was coded as 1, while “indifferent” and “do not need” were coded as 0. We combined “indifferent” and “do not need” into a single reference category because both represent the absence of expressed need, providing a conservative estimate of education need.

Perceived necessity of school-based reproductive health courses was assessed by asking: “Should schools offer reproductive health courses?” Response options were “necessary” and “not necessary.” “Necessary” was coded as 1 and “not necessary” as 0.

#### 2.3.4 Outcome: desired education topics

Desired reproductive health education topics were assessed using a multiple-choice question: “What reproductive health topics would you like to learn about?” Options included: healthy sexual behavior, prevention of sexually transmitted diseases and gynecological diseases, contraception knowledge, reproductive anatomy and basic function, sexual dysfunction, infertility treatment, sexual psychology, sexual ethics, sexual physiology and development, and “I do not want to learn any knowledge.” Multiple selections were allowed. We conducted a logic check on responses to the “do not want to learn any knowledge” option: among the 1,760 participants who selected this option, 1,688 (95.91%) also selected at least one specific topic (Supplementary Table S4). This indicates that the “no topic” response was not a valid indicator of no educational need. Therefore, this response option was retained for descriptive transparency only and was not used as an outcome in any regression analysis, nor was it used to recode the separate education-need outcome (Q9), as these are distinct constructs.

#### 2.3.5 Covariates

Covariates included age, educational level (junior college, undergraduate, postgraduate), household registration type (rural vs. urban, based on hukou origin rather than current residence), only-child status (yes vs. no), academic major (medical/health-related vs. non-medical), and main financial source (parental support, part-time employment, student loan, scholarship, support from relatives or friends, and other). These variables were selected *a priori* because they may be associated with both the primary source of reproductive health information and the study outcomes. Participation in school-based reproductive health education (Q8: never/occasionally/frequently) was not included as a covariate in the main models because it is conceptually related to the exposure (school education as primary source) and may lie on the causal pathway. The temporal relationship between Q8 (past participation) and Q11 (current primary source) cannot be determined in this cross-sectional design; Q8 may act as either a confounder or a mediator. Therefore, Q8 was examined in a separate sensitivity analysis (Model 3, Supplementary Table S3) and the results are interpreted as conditional associations rather than causal effects. Detailed variable definitions and coding are provided in Supplementary Table S1.

### 2.4 Statistical analysis

Descriptive statistics were used to summarize information sources, contraceptive familiarity, education needs, and desired topics. Categorical variables were presented as frequencies and percentages. Chi-square tests were used to compare categorical outcomes across information source groups. Cramer's V was used to describe the strength of association.

Multivariable binary logistic regression models were constructed to examine the adjusted associations between primary information source and three outcomes: higher self-reported contraceptive familiarity, need for reproductive health education, and perceived necessity of school-based reproductive health courses. School education was used as the reference group. To account for the clustered nature of the data (students nested within 24 universities), we estimated university-level clustered robust standard errors in all regression models. With 24 clusters, the estimates are expected to be conservative; we have added a cautionary note in the limitations section regarding the modest number of clusters. Department- or faculty-level clustering was not modeled due to the absence of these identifiers in the de-identified dataset, which is acknowledged as a limitation. The main models were adjusted for age, educational level, household registration type, academic major, main financial source, and only-child status. Participation in school-based reproductive health education was not included in the main adjustment set due to its conceptual overlap with the exposure and potential role as a mediating variable on the causal pathway; it was examined in a separate sensitivity model (Model 3).

In sensitivity analyses, we conducted ordinal logistic regression using the full four-level contraceptive familiarity outcome (very familiar, generally familiar, not very familiar, do not understand at all). Although the Brant test indicated a statistically significant violation of the proportional odds assumption (*P* < 0.001), which is expected given the large sample size (*N* = 6,428), ordinal logistic regression was retained as the primary sensitivity analysis because it preserves the ordered nature of the outcome and is robust to modest violations in large samples. Multinomial logistic regression results are also presented (Supplementary Table S5).

We report three models for each outcome: Model 1 (crude): unadjusted associations; Model 2 (main adjusted): adjusted for age, educational level, household registration, academic major, economic source, and only-child status; Model 3 (sensitivity): additionally adjusted for participation in school-based reproductive health education.

Adjusted odds ratios (aORs), 95% confidence intervals (CIs), and *P*-values are reported for Model 2. A two-sided *P*-value <0.05 was considered statistically significant. The three outcomes (higher self-reported contraceptive familiarity, perceived need for reproductive health education, and perceived necessity of school-based courses) were prespecified. Given the exploratory nature of the study and the prespecified outcomes, no formal adjustment for multiple comparisons was applied. We emphasize that marginal *P*-values should be interpreted with caution; primary emphasis is placed on effect sizes and confidence intervals rather than statistical significance. All findings should be considered hypothesis-generating and require confirmation in future studies. All analyses were performed using R version 4.3.1 (R Foundation for Statistical Computing).

Complete regression coefficients are presented in Supplementary Table S2; sensitivity analyses adjusting for school-based education participation are shown in Supplementary Table S3 and Supplementary Figure S1.

### 2.5 Ethical considerations

The study was approved by the Ethics Committee of Shenzhen Hospital of Integrated Traditional Chinese and Western Medicine (KY-2023-033-02), the Ethics Committee of Pingshan District Central Hospital (PZXY-EC-AP-020) and the Ethics Committee of Heze Municipal Hospital (2024-KY003-06). Electronic informed consent was obtained from all participants before completion of the questionnaire. The study was conducted in accordance with the Declaration of Helsinki.

## 3 Results

### 3.1 Participant characteristics by primary information source

A total of 6,428 FCSs were included. The median age was 23 years [interquartile range, 21–26 years; mean (SD), 23.29 (3.38) years]. The primary information source was associated with age (Kruskal–Wallis *H* = 469.11, *P* < 0.001; η^2^ = 0.073), with students reporting school education as their primary source being older (median, 26 years) than those in the other source groups (medians, 22–23 years). Educational level, household registration origin, only-child status, academic major, main financial support, and school-based education participation also differed across information-source groups (all *P* ≤ 0.001), although the corresponding Cramer's *V* values were small (0.052–0.092; Table 1).

**Table 1:** Characteristics of participants according to the primary source of reproductive health information.

Characteristic	Internet	School education	Popular science books	Family education	Peers/ friends	Radio/ television	*P* value
Age, median (IQR), y	22 (20-24)	26 (23-28)	22 (20-25)	23 (21-27)	22 (20-25)	22 (20-25)	<0.001
Educational level							<0.001
Junior college	623 (35.5)	340 (24.6)	375 (32.3)	282 (31.4)	290 (36.1)	143 (33.2)	
Undergraduate	765 (43.6)	603 (43.7)	547 (47.1)	381 (42.4)	335 (41.7)	196 (45.5)	
Master's degree or above	366 (20.9)	437 (31.7)	240 (20.7)	235 (26.2)	178 (22.2)	92 (21.3)	
Household registration origin							<0.001
Urban	872 (49.7)	569 (41.2)	641 (55.2)	407 (45.3)	377 (46.9)	215 (49.9)	
Rural	882 (50.3)	811 (58.8)	521 (44.8)	491 (54.7)	426 (53.1)	216 (50.1)	
Only child							<0.001
Yes	884 (50.4)	786 (57.0)	639 (55.0)	468 (52.1)	401 (49.9)	211 (49.0)	
No	870 (49.6)	594 (43.0)	523 (45.0)	430 (47.9)	402 (50.1)	220 (51.0)	
Academic major							0.001
Medicine/health sciences-related	890 (50.7)	652 (47.2)	554 (47.7)	466 (51.9)	441 (54.9)	238 (55.2)	
Non-medicine/health sciences-related	864 (49.3)	728 (52.8)	608 (52.3)	432 (48.1)	362 (45.1)	193 (44.8)	
Main financial support							<0.001
Parental support	1086 (61.9)	733 (53.1)	690 (59.4)	488 (54.3)	438 (54.5)	217 (50.3)	
Part-time employment	263 (15.0)	284 (20.6)	183 (15.7)	153 (17.0)	123 (15.3)	76 (17.6)	
Student loan	162 (9.2)	118 (8.6)	109 (9.4)	94 (10.5)	88 (11.0)	48 (11.1)	
Scholarship	120 (6.8)	97 (7.0)	98 (8.4)	72 (8.0)	85 (10.6)	46 (10.7)	
Support from relatives or friends	104 (5.9)	109 (7.9)	70 (6.0)	74 (8.2)	60 (7.5)	39 (9.0)	
Other	19 (1.1)	39 (2.8)	12 (1.0)	17 (1.9)	9 (1.1)	5 (1.2)	
School education participation							<0.001
Participated	706 (40.3)	650 (47.1)	531 (45.7)	390 (43.4)	342 (42.6)	157 (36.4)	
Did not participate	516 (29.4)	413 (29.9)	349 (30.0)	250 (27.8)	233 (29.0)	135 (31.3)	
School did not offer activities	532 (30.3)	317 (23.0)	282 (24.3)	258 (28.7)	228 (28.4)	139 (32.3)	

Data are *n* (%) unless otherwise stated. Age was compared using the Kruskal–Wallis test; categorical variables were compared using Pearson's chi-square test. Information-source group sizes were Internet, *n* = 1,754; School education, *n* = 1,380; Popular science books, *n* = 1,162; Family education, *n* = 898; Peers/friends, *n* =803; and Radio/television, *n* = 431.

### 3.2 Primary information sources and unadjusted outcome distributions

The Internet was the most frequently reported primary source of reproductive health information (*n* = 1,754, 27.29%), followed by school education (*n* = 1,380, 21.47%) and popular science books (*n* = 1,162, 18.08%; Figure 1). The distribution of self-reported contraceptive familiarity differed across information-source groups (χ^2^ = 96.10, df = 15, *P* < 0.001; Cramer's *V* = 0.071). The school-education group had the highest proportion reporting higher self-reported contraceptive familiarity (69.71%), whereas peers/friends (57.41%) and radio/television (57.54%) had the lowest proportions. Perceived need for reproductive health education and the view that a school course was necessary also differed by source (both *P* < 0.001); effect sizes were small (Cramer's *V* = 0.084 and 0.120, respectively; Table 2). The full four-level familiarity distribution is shown in Figure 2.

**Figure 1:**
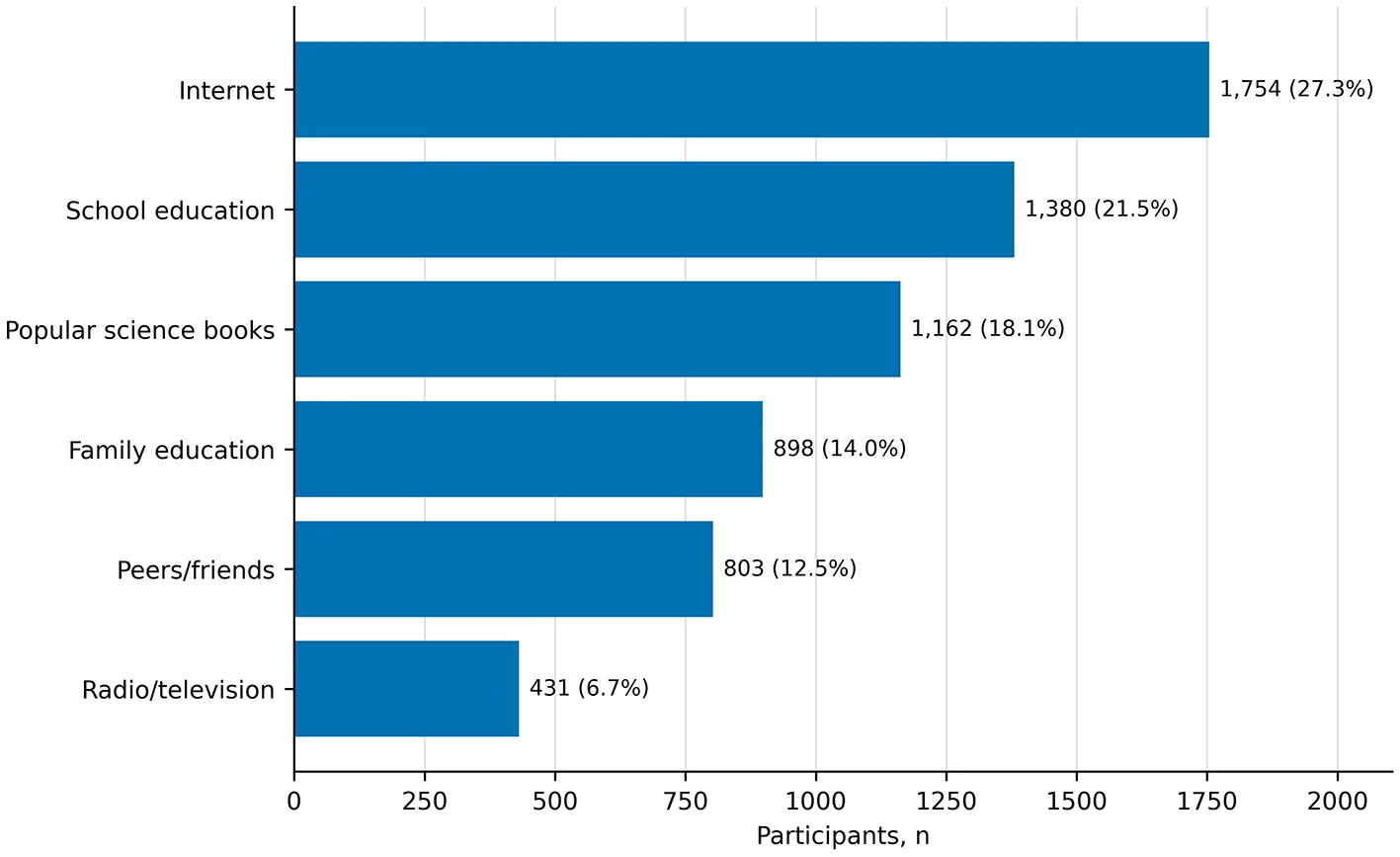
Primary sources of reproductive health information among female college students. Bars are ordered by frequency and labels show *N* (% of *N* = 6,428).

**Table 2:** Primary information source and unadjusted distributions of study outcomes.

Outcome	Internet	School education	Popular science books	Family education	Peers/ friends	Radio/ television	χ^2^ (df)	*P* value	Cramer's *V*
Self-reported familiarity									
Very familiar	527 (30.0)	411 (29.8)	318 (27.4)	264 (29.4)	237 (29.5)	128 (29.7)	96.10 (15)	<0.001	0.071
Generally familiar	592 (33.8)	551 (39.9)	456 (39.2)	273 (30.4)	224 (27.9)	120 (27.8)			
Not very familiar	335 (19.1)	264 (19.1)	223 (19.2)	192 (21.4)	164 (20.4)	94 (21.8)			
Self-reported familiarity: Do not understand at all	300 (17.1)	154 (11.2)	165 (14.2)	169 (18.8)	178 (22.2)	89 (20.6)			
Higher self-reported contraceptive familiarity	1119 (63.8)	962 (69.7)	774 (66.6)	537 (59.8)	461 (57.4)	248 (57.5)	52.57 (5)	<0.001	0.090
Perceived need for reproductive health education	912 (52.0)	782 (56.7)	535 (46.0)	463 (51.6)	357 (44.5)	206 (47.8)	45.15 (5)	<0.001	0.084
School course considered necessary	1294 (73.8)	1100 (79.7)	803 (69.1)	601 (66.9)	531 (66.1)	267 (61.9)	93.26 (5)	<0.001	0.120

Data are *n* (% within each information-source group). Higher self-reported contraceptive familiarity was defined as reporting either “very familiar” or “generally familiar” with contraceptive methods. Pearson's chi-square tests were used.

**Figure 2:**
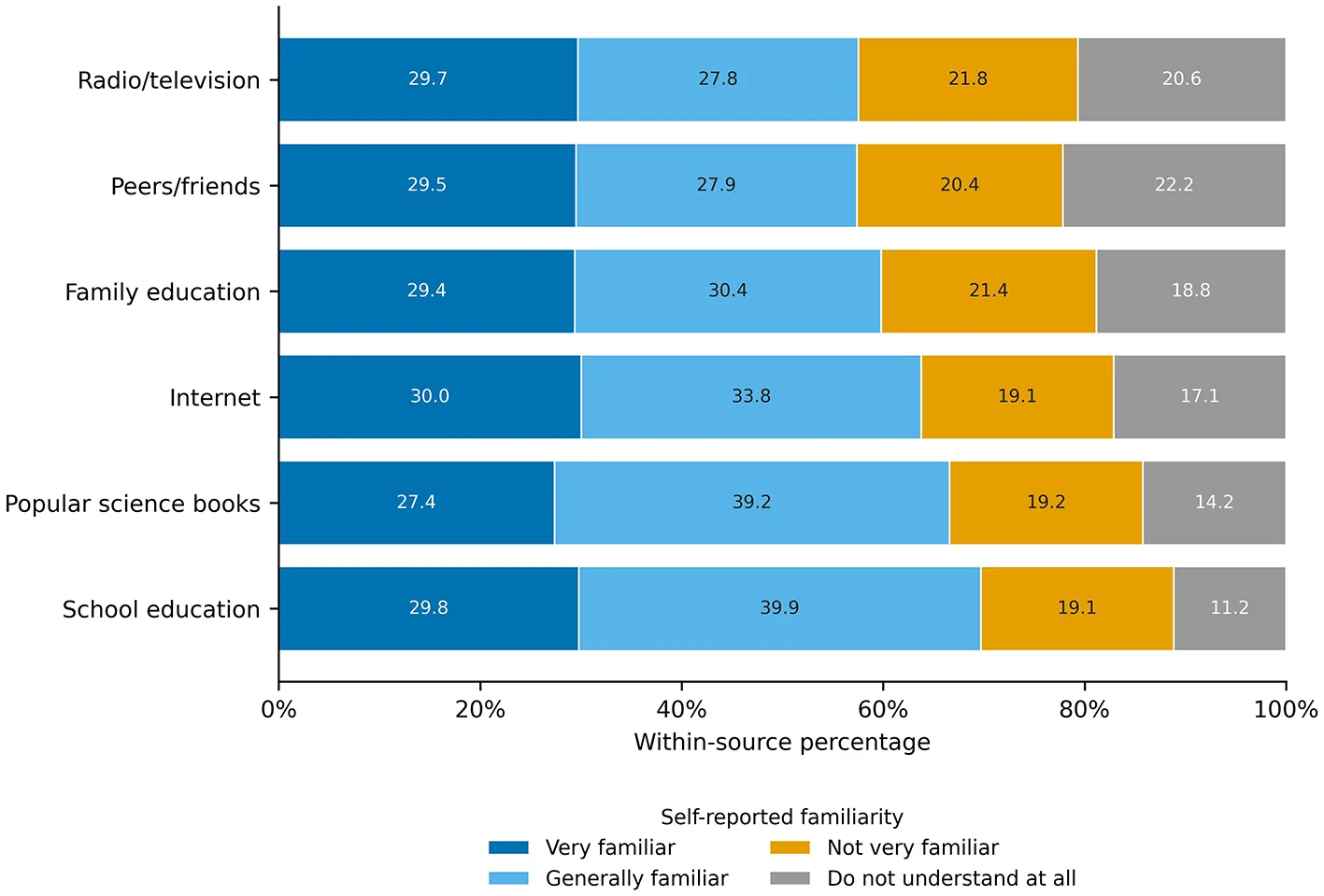
Distribution of self-reported familiarity with contraceptive methods by primary information source. Bars show within-source percentages. Self-reported familiarity differed across source groups (χ^2^ = 96.10, df = 15, *P* < 0.001; Cramer's *V* = 0.071).

### 3.3 Preferred reproductive health education topics

The most frequently selected education topic was prevention of sexually transmitted infections and gynecological diseases (*n* = 4,769, 74.19%), followed by healthy sexual behavior (*n* = 4,558, 70.91%) and contraception (*n* = 4,444, 69.14%). Participants also commonly selected reproductive anatomy and basic function (61.34%) and sexual physiology and development (56.24%). Because multiple responses were allowed, topic percentages do not sum to 100% (Table 3). Supplementary Table S4 presents the cross-tabulation of the “does not wish to learn any topic” response with selection of specific topics.

**Table 3:** Preferred reproductive health education topics among FCSs (*N* = 6,428).

Preferred education topic	*N*	%
Prevention of sexually transmitted infections and gynecological diseases	4,769	74.19
Healthy sexual behavior	4,558	70.91
Contraception	4,444	69.14
Reproductive anatomy and basic function	3,943	61.34
Sexual physiology and development	3,615	56.24
Sexual ethics	3,133	48.74
Sexual psychology	3,060	47.60
Sexual dysfunction	2,541	39.53
Infertility treatment	2,046	31.83
Does not wish to learn any topic	1,760	27.38

Multiple responses were allowed; percentages use the total analytic sample as the denominator. The “Does not wish to learn any topic” response is presented as originally recorded; however, 1,688 of 1,760 respondents (95.91%) who selected this option also selected at least one specific topic. Therefore, this response should not be interpreted as a definitive absence of educational need.

### 3.4 Associations between information source and study outcomes

In crude analyses, Internet, family education, peers/friends, and radio/television were each associated with lower odds of higher self-reported contraceptive familiarity than school education. After adjustment for age, educational level, household registration origin, only-child status, academic major, and main financial support, with university-level clustered standard errors, the Internet was no longer statistically different from school education (aOR = 0.951, 95% CI: 0.811–1.114). Family education (aOR = 0.744, 95% CI: 0.621–0.892), peers/friends (aOR = 0.734, 95% CI: 0.608–0.885), and radio/television (aOR = 0.733, 95% CI: 0.582–0.922) remained associated with lower odds of higher self-reported contraceptive familiarity. Popular science books did not differ significantly from school education (aOR = 1.042, 95% CI: 0.875–1.240). However, the absence of a statistically significant difference should not be interpreted as equivalence, and the confidence intervals include values that would be clinically or educationally meaningful. For perceived need for reproductive health education, only peers/friends (aOR = 0.777, 95% CI: 0.648–0.932) and popular science books (aOR = 0.821, 95% CI: 0.697–0.967) showed lower adjusted odds relative to school education. For considering a school course necessary, family education (aOR = 0.641, 95% CI: 0.525–0.782), peers/friends (aOR = 0.701, 95% CI: 0.570–0.861), popular science books (aOR = 0.763, 95% CI: 0.630–0.923), and radio/television (aOR = 0.583, 95% CI: 0.456–0.745) showed lower adjusted odds; the Internet (aOR = 1.012, 95% CI: 0.846–1.211) did not differ significantly from school education. Complete crude and adjusted estimates are reported in Table 4 and summarized visually in Figure 3. Full regression outputs and sensitivity analyses are available in Supplementary Table S2, Supplementary Table S3, and Supplementary Figure S1.

**Table 4:** Crude and adjusted associations between primary reproductive health information source and study outcomes.

Outcome	Primary source vs. school education	Crude OR (95% CI)	*P* value	aOR (95% CI)	*P* value
Higher self-reported contraceptive familiarity	Internet	0.766 (0.659–0.890)	<0.001	0.951 (0.811–1.114)	0.530
	Popular science books	0.867 (0.733–1.025)	0.094	1.042 (0.875–1.240)	0.647
	Family education	0.646 (0.542–0.771)	<0.001	0.744 (0.621–0.892)	0.001
	Peers/friend	0.586 (0.489–0.702)	<0.001	0.734 (0.608–0.885)	0.001
	Radio/television	0.589 (0.471–0.736)	<0.001	0.733 (0.582–0.922)	0.008
Perceived need for reproductive health education	Internet	0.828 (0.719–0.954)	0.009	1.067 (0.919–1.239)	0.396
	Popular science books	0.653 (0.558–0.763)	<0.001	0.821 (0.697–0.967)	0.018
	Family education	0.814 (0.688–0.964)	0.017	0.957 (0.804–1.138)	0.617
	Peers/friend	0.612 (0.514–0.729)	<0.001	0.777 (0.648–0.932)	0.006
	Radio/television	0.700 (0.564–0.870)	0.001	0.908 (0.726–1.136)	0.400
School course considered necessary	Internet	0.716 (0.605–0.848)	<0.001	1.012 (0.846–1.211)	0.895
	Popular science books	0.569 (0.475–0.682)	<0.001	0.763 (0.630–0.923)	0.005
	Family education	0.515 (0.425–0.624)	<0.001	0.641 (0.525–0.782)	<0.001
	Peers/friend	0.497 (0.408–0.605)	<0.001	0.701 (0.570–0.861)	<0.001
	Radio/television	0.414 (0.328–0.524)	<0.001	0.583 (0.456–0.745)	<0.001

School education was the reference category. Adjusted models included age, educational level, household registration origin, only-child status, academic major, and main financial support. Participation in school-based reproductive health education was not adjusted for in the primary model because of potential overadjustment. All models included N = 6,428 participants.

**Figure 3:**
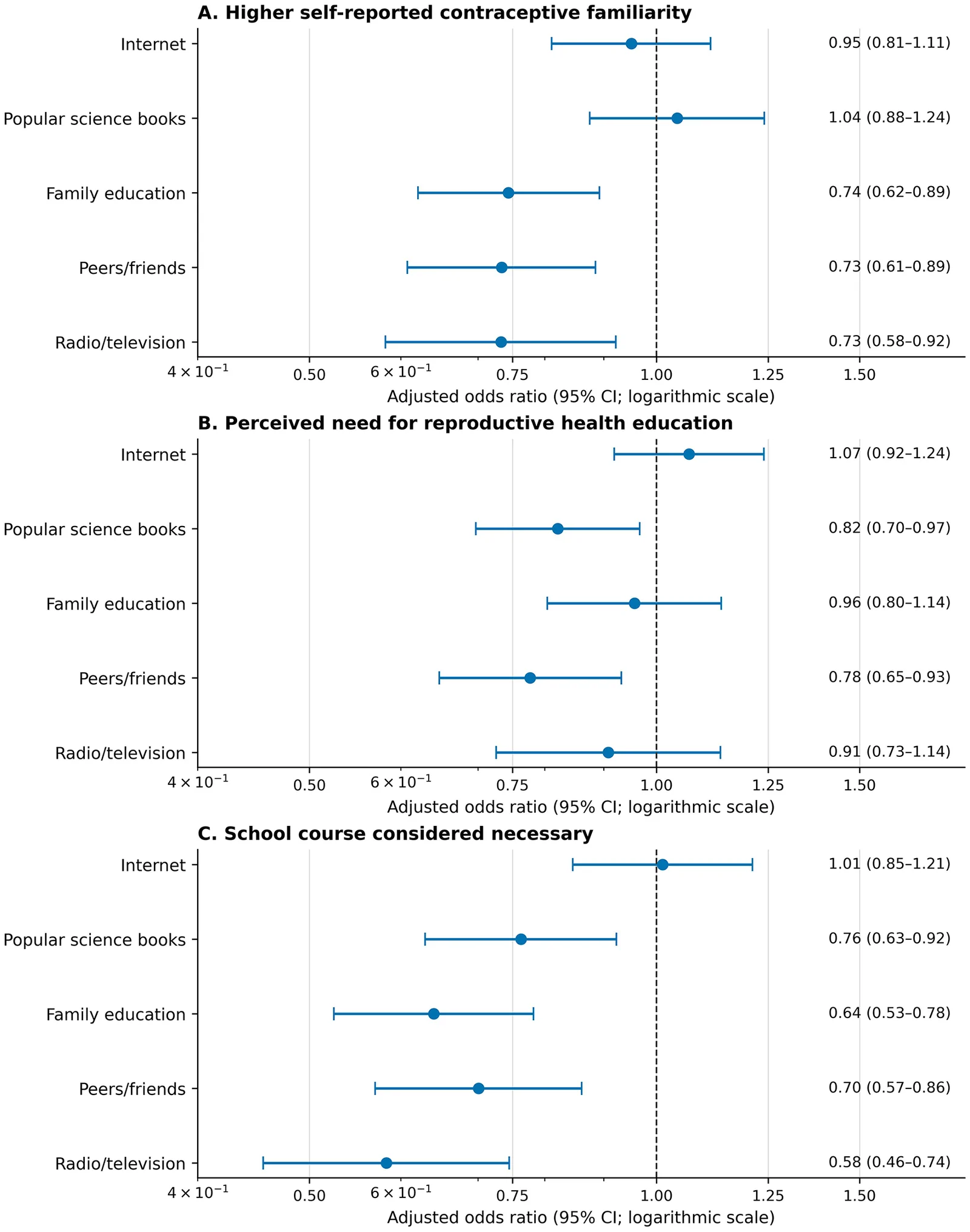
Adjusted associations between primary information source and three reproductive health outcomes. Points represent adjusted odds ratios and horizontal bars represent 95% confidence intervals. School education is the reference category. Models adjust for age, educational level, household registration origin, only-child status, academic major, and main financial support.The vertical dashed line denotes an odds ratio of 1. The horizontal axis is logarithmically scaled.

### 3.5 Adjusted associations of participant characteristics with study outcomes

Age was positively associated with all three outcomes: higher self-reported contraceptive familiarity (aOR per year = 1.075, 95% CI 1.056–1.094), perceived need for reproductive health education (aOR = 1.112, 95% CI 1.094–1.131), and considering a school course necessary (aOR = 1.169, 95% CI 1.146–1.193). The complete covariate estimates are provided in Table 5 to make model adjustment transparent. The modest Nagelkerke *R*^2^ values (0.057, 0.056, and 0.105, respectively) indicate that information source and included covariates explained a limited proportion of outcome variation. This suggests that other factors beyond information source, such as individual health literacy, motivation, past sexual education, and access to services, are important determinants of contraceptive familiarity and education needs.

**Table 5:** Adjusted associations of participant characteristics with the three study outcomes.

Characteristic	Higher familiarity aOR (95% CI)	*P*	Education need aOR (95% CI)	*P*	Course necessary aOR (95% CI)	*P*
Age, per 1-year increase	1.075 (1.056–1.094)	<0.001	1.112 (1.094–1.131)	<0.001	1.169 (1.146–1.193)	<0.001
Educational level: junior college vs. undergraduate	0.794 (0.703–0.898)	<0.001	1.240 (1.101–1.398)	<0.001	0.978 (0.857–1.117)	0.747
Educational level: master's degree or above vs. undergraduate	0.940 (0.820–1.077)	0.370	1.161 (1.021–1.321)	0.023	0.940 (0.810–1.091)	0.413
Rural vs. urban household registration origin	0.994 (0.895–1.103)	0.903	1.105 (0.999–1.222)	0.053	0.941 (0.840–1.055)	0.298
Only child: yes vs. no	1.156 (1.041–1.285)	0.007	1.120 (1.012–1.240)	0.028	1.198 (1.069–1.343)	0.002
Medicine/health sciences-related vs. non-related major	0.708 (0.637–0.788)	<0.001	0.919 (0.829–1.019)	0.108	0.850 (0.757–0.955)	0.006
Financial support: other vs. parental support	1.210 (0.765–1.914)	0.414	1.009 (0.668–1.525)	0.965	1.322 (0.739–2.366)	0.347
Financial support: part-time employment vs. parental support	1.027 (0.886–1.189)	0.726	0.879 (0.764–1.010)	0.069	0.730 (0.624–0.855)	<0.001
Financial support: scholarship vs. parental support	0.701 (0.580–0.847)	<0.001	0.750 (0.620–0.907)	0.003	0.560 (0.459–0.682)	<0.001
Financial support: student loan vs. parental support	0.844 (0.707–1.009)	0.062	0.818 (0.687–0.974)	0.024	0.740 (0.612–0.894)	0.002
Financial support: relatives/friends vs. parental support	1.134 (0.916–1.403)	0.248	0.879 (0.720–1.073)	0.204	0.688 (0.551–0.858)	<0.001

Estimates are from the same multivariable models as in Table 4. The source-of-information coefficients are displayed in Table 4. Reference categories are undergraduate education, urban household registration origin, not being an only child, a non-medicine/health sciences-related major, and parental financial support.

### 3.6 Education-topic preferences by primary information source

Topic preferences varied across information-source groups, although these descriptive subgroup patterns should not be interpreted as causal effects. Prevention of sexually transmitted infections and gynecological diseases, healthy sexual behavior, and contraception were the three most commonly selected topics in every source group. The topic-by-source matrix is presented in Table 6 and visualized in Figure 4 to support planning of targeted education content. The distribution of school-based education participation by source is presented separately in Figure 5.

**Table 6:** Preferred reproductive health education topics according to primary information source.

Preferred education topic	Internet	School education	Popular science books	Family education	Peers/ friends	Radio/ television
Prevention of sexually transmitted infections and gynecological diseases	1200 (68.4)	1156 (83.8)	803 (69.1)	682 (75.9)	628 (78.2)	300 (69.6)
Healthy sexual behavior	1142 (65.1)	1031 (74.7)	804 (69.2)	660 (73.5)	599 (74.6)	322 (74.7)
Contraception	1069 (60.9)	1085 (78.6)	750 (64.5)	631 (70.3)	602 (75.0)	307 (71.2)
Reproductive anatomy and basic function	1026 (58.5)	876 (63.5)	675 (58.1)	583 (64.9)	523 (65.1)	260 (60.3)
Sexual physiology and development	938 (53.5)	943 (68.3)	587 (50.5)	493 (54.9)	428 (53.3)	226 (52.4)
Sexual ethics	827 (47.1)	684 (49.6)	566 (48.7)	462 (51.4)	372 (46.3)	222 (51.5)
Sexual psychology	795 (45.3)	783 (56.7)	490 (42.2)	434 (48.3)	376 (46.8)	182 (42.2)
Sexual dysfunction	689 (39.3)	532 (38.6)	435 (37.4)	365 (40.6)	340 (42.3)	180 (41.8)
Infertility treatment	558 (31.8)	418 (30.3)	356 (30.6)	303 (33.7)	263 (32.8)	148 (34.3)
Does not wish to learn any topic	437 (24.9)	267 (19.3)	371 (31.9)	288 (32.1)	254 (31.6)	143 (33.2)

Data are *n* (% within each information-source group). Multiple responses were allowed. The final row reflects the original response option and has known logical overlap with selected specific topics (see Table 3 note); it should be interpreted with caution.

**Figure 4:**
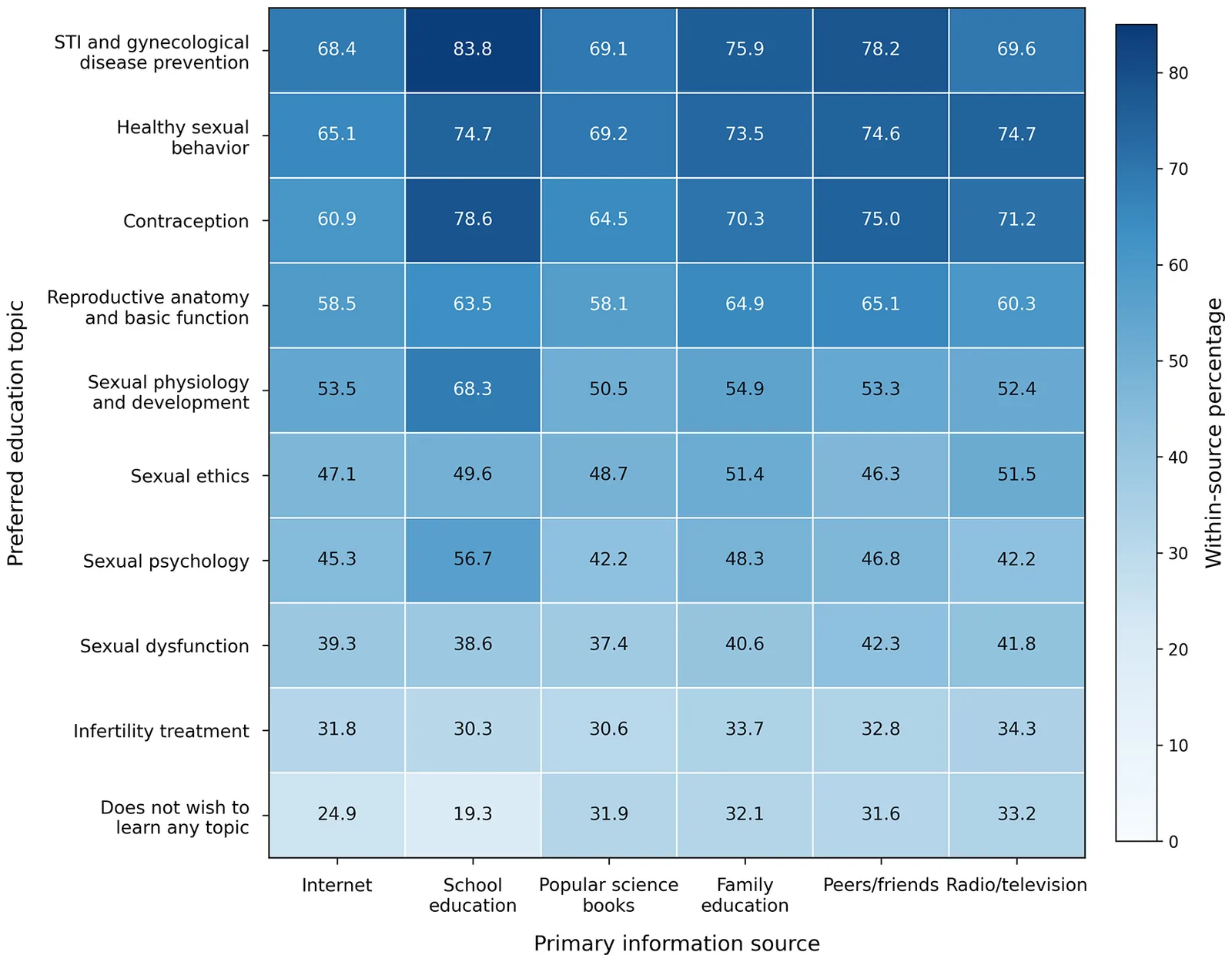
Preferred reproductive health education topics according to primary information source. Each cell displays the percentage of students within an information-source group selecting the topic. Multiple responses were allowed; the color scale represents the within-source percentage.

**Figure 5:**
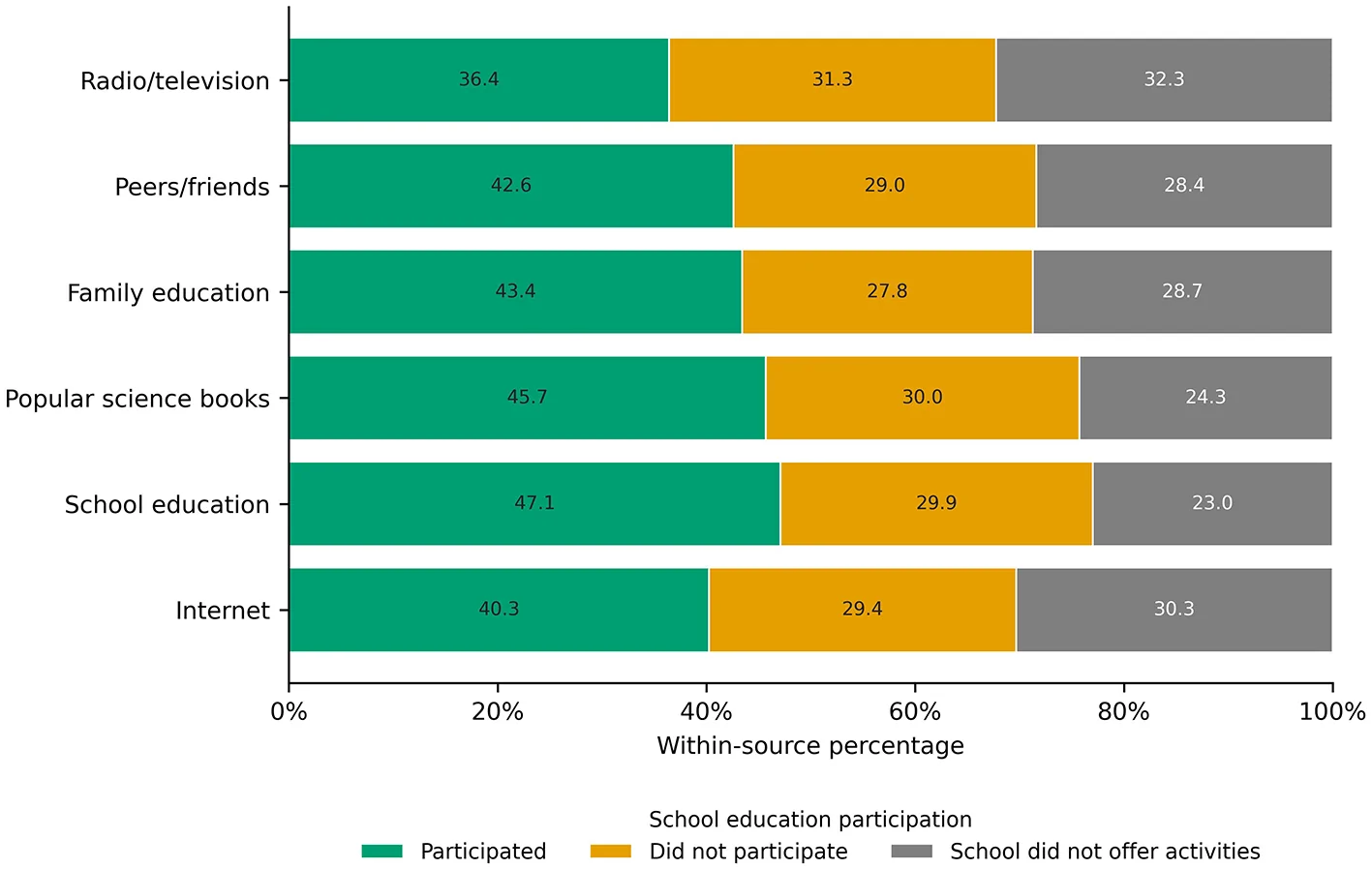
School-based reproductive health education participation by primary information source. Bars show within-source percentages for participation status. This figure is descriptive and illustrates the conceptual overlap between school education as an information source and prior participation in school-based education activities.

## 4 Discussion

This cross-sectional study examined the relationship between reproductive health information sources, self-reported contraceptive familiarity, and reproductive health education needs among 6,428 FCSs from 24 universities in China. Consistent with the cross-sectional design, all associations reported herein should be interpreted as correlational rather than causal. The Internet was the most frequently reported primary source of reproductive health information, followed by school education and popular science books.However, students whose main source was school education had the highest proportion of higher self-reported contraceptive familiarity and the strongest perceived need for school-based reproductive health courses. After adjustment for age, educational level, household registration origin, only-child status, academic major, and main financial support, with university-level clustered standard errors, family education, peers/friends, and radio/television were associated with lower odds of higher self-reported contraceptive familiarity compared with school education (25). The Internet and popular science books did not differ significantly from school education in adjusted analyses (30–32).

The finding that the Internet was the leading information source reflects the central role of digital media in young women's health information seeking (33). Online platforms are accessible, private, and convenient, which may be particularly important for topics such as contraception and sexual health (34). Although Internet use was the most frequently reported primary information source, the adjusted odds of higher self-reported contraceptive familiarity did not differ significantly between students relying primarily on the Internet and those relying on school education (35). This finding suggests that the crude difference may partly reflect differences in participant characteristics across source groups, including age and educational background. Nevertheless, the cross-sectional design and the self-reported outcome do not permit conclusions regarding the effectiveness or quality of Internet-based information. However, the absence of a statistically significant difference should not be interpreted as equivalence, and the confidence interval (0.811–1.114) includes values that would be clinically or educationally meaningful.

School education was associated with a more favorable pattern in this sample. Students relying primarily on school education had the lowest proportion reporting no contraceptive familiarity and the highest proportion with higher self-reported contraceptive familiarity (36). They also showed the strongest endorsement of school-based reproductive health courses.

This finding may reflect a dual role of school education: it may be associated with more structured knowledge acquisition and may also be related to increased awareness of unmet learning needs (37). However, the higher education need reported by the school education group (56.67%) should not be interpreted unidirectionally as a positive effect. Alternative explanations include:school education may heighten awareness of knowledge gaps, students who have received some education may better recognize what they do not know, current school-based education may be insufficient to meet students' needs, or students with higher health consciousness may both seek out school education and report higher educational needs (38). These competing interpretations cannot be disentangled with the current cross-sectional data and should be explored in future longitudinal or mixed-methods research. Consistent with the cross-sectional design, these associations should be interpreted as correlational rather than as evidence of causal effects.

Popular science books performed relatively well in relation to contraceptive familiarity (39). In the adjusted model, students relying on popular science books did not differ significantly from those relying on school education in higher self-reported contraceptive familiarity (40). This may reflect the relatively structured and edited nature of printed or formal popular science materials. However, students relying on popular science books had lower odds of reporting reproductive health education need and lower odds of considering school-based courses necessary, possibly because they perceived their existing self-study as sufficient (41). We further examined the four-level familiarity outcome using ordinal and multinomial logistic regression (Supplementary Table S4). The multinomial results showed that the associations seen in the binary analysis came mainly from the difference between “generally familiar” and “do not understand,” with less difference at the “very familiar” threshold. This suggests that the binary outcome captures the main pattern, though the effect is stronger at the lower end of familiarity, students who relied on informal sources were more likely to report little or no familiarity than moderate familiarity.

Informal interpersonal sources, particularly peers or friends, were consistently associated with less favorable outcomes. Students relying on peers or friends had lower odds of higher self-reported contraceptive familiarity and lower odds of reporting education need or course necessity compared with those relying on school education (42). Peer communication may be an important channel for emotional support and experience sharing, but it may not reliably provide accurate or comprehensive reproductive health information (43).

Family education occupied an intermediate position. Although family education can provide early and trusted guidance, students relying primarily on family education had lower odds of higher self-reported contraceptive familiarity compared with those relying on school education (44). This may reflect variability in parental knowledge, communication comfort, cultural norms, and willingness to discuss contraception or sexual health (45).

The analysis of desired education topics showed that students' needs were highly practical. Prevention of sexually transmitted diseases and gynecological diseases, healthy sexual behavior, and contraception knowledge were the most frequently selected topics (46). These results indicate that FCSs are not only interested in general reproductive biology, but also in content directly related to disease prevention, sexual decision-making, and self-protection.

The relatively small effect sizes (Cramer's *V* = 0.071 for the overall chi-square test of contraceptive familiarity by information source) warrant attention. Although the differences reached statistical significance due to the large sample size, the practical significance of these differences may be modest. This underscores the importance of interpreting the findings as associations deserving further investigation rather than as strong determinants of contraceptive familiarity.

This study has implications for campus reproductive health services. School-based reproductive health education should remain a core component of student health promotion, even in an environment where online information is widely accessible (47, 48). Digital resources should not be viewed as a replacement for school education (49). Instead, universities could integrate curated digital materials into formal courses and guide students toward reliable online resources. Interventions to improve digital health literacy—including skills to evaluate the credibility of online reproductive health information—may be particularly valuable for students who rely primarily on the Internet (6, 50, 51).

Several limitations should be considered. First, the cross-sectional design precludes causal inference. The associations observed may be bidirectional or explained by unmeasured confounding. Second, all variables were self-reported, and contraceptive familiarity was measured by perceived familiarity rather than objective knowledge testing. This measure does not assess knowledge of contraceptive efficacy, correct usage, or side-effect profiles; nor can its criterion validity be evaluated with the present dataset. The single-item approach may also obscure method-specific knowledge differences. Third, the multiple-response item concerning preferred education topics contained a logical inconsistency: most participants (95.91%) who selected “Does not wish to learn any topic” also selected at least one specific topic (Supplementary Table S4). This response option was retained as originally recorded for descriptive transparency but was not interpreted as a valid indicator of no educational need, nor was it used to recode the separate education-need outcome. Fourth, information source was measured as the primary source only; students may use multiple sources simultaneously, and the study could not capture the quality, frequency, or depth of exposure to each source. Fifth, the convenience sampling approach limits generalizability; the sample is not representative of all FCSs in China or any participating province. The 12 provinces were selected for geographic diversity but do not constitute a probability sample of Chinese regions. Findings should be extrapolated with caution to populations with different demographic, cultural, or educational contexts. Sixth, several potentially important confounders, including sexual experience, relationship status, parental education, media literacy, socioeconomic status, and access to health services, were not measured and may have influenced both information source and familiarity. These unmeasured variables represent potential sources of residual confounding. Seventh, “school education” is a heterogeneous exposure; the content, frequency, format, and quality of school-based reproductive health education were not characterized in this study. Future research should collect detailed information on school education characteristics to better understand what aspects of school education may drive the observed associations. Eighth, FCSs were nested within 24 universities, which we addressed using clustered robust standard errors; this approach adjusts for within-school correlation in a reduced-form manner and cannot fully account for all school-level or department-level unmeasured heterogeneity. Ninth, the primary analysis did not adjust for multiple comparisons; while the outcomes were prespecified, marginal *P*-values should be interpreted as exploratory evidence rather than definitive significance.

Despite these limitations, the study contributes to reproductive health education research by shifting attention from whether students have received education to where they primarily obtain reproductive health information. The findings show that information source is associated with both contraceptive familiarity and educational demand in this large multi-province sample.

## 5 Conclusions

In this multi-province convenience sample of FCSs in China, the Internet was the most common primary source of reproductive health information, but school education was associated with the most favorable profile of self-reported contraceptive familiarity and education demand. Compared with school education, reliance on family education, peers or friends, and radio or television was associated with lower odds of higher self-reported contraceptive familiarity; the Internet and popular science books did not differ significantly from school education in adjusted analyses. However, the modest effect sizes and low explanatory power indicate that information source alone explains a limited proportion of outcome variation. Students expressed strong interest in practical topics, especially prevention of sexually transmitted diseases and gynecological diseases, healthy sexual behavior, and contraception knowledge. These findings suggest that school-based reproductive health education remains relevant in the digital information environment. Universities should consider strengthening structured reproductive health curricula, integrating reliable online resources, and providing targeted support for students who rely mainly on informal or less structured information sources. However, given the cross-sectional and convenience-sampling design, and the modest effect sizes observed, these findings should be interpreted as associations requiring further evaluation in longitudinal and intervention studies rather than as evidence of strong causal effects or a basis for immediate policy change.

## Data Availability

The raw data supporting the conclusions of this article will be made available by the authors, without undue reservation.
