# Supplementary material for: Sources of reproductive health information and their associations with self-reported contraceptive familiarity and education needs among female college students: a cross-sectional study in China
Source: Front Public Health. 2026 Sep 18;14:1949119. doi: 10.3389/fpubh.2026.1949119 (PMC13630764; doi:10.3389/fpubh.2026.1949119)
Supplement: Supplementary file 1 [file Data_Sheet_1.docx]

**Supplementary Materials**

*Sources of Reproductive Health Information and Their Associations With Self-Reported Contraceptive Familiarity and Education Needs Among Female College Students: A Cross-Sectional Study in China*

# Supplementary Table S1

**Supplementary Table S1. Questionnaire variables, original Chinese items, response options, coding for analysis, and analytic variable names**

| **Item** | **Construct** | **OriginalChineseitem** | **Responseoptions** | **Codingforanalysis** | **Analyticvariable** |
| --- | --- | --- | --- | --- | --- |
| Q1 | Age | 1、您的年龄:___周岁。 | Age in completed years | Continuous, per 1-year increase | Age |
| Q2 | Educational level | 2、您现在就读的年级? | Junior college; Undergraduate; Master's degree or above | Dummy variables; undergraduate reference | Educational level |
| Q3 | Household registration origin | 3、您的原户籍所在地: | Urban; Rural | Rural=1; urban reference | Household registration origin |
| Q4 | Only-child status | 4、您是独生子女吗? | Yes; No | Yes=1; no reference | Only child |
| Q5 | Academic major | 5、您所学专业是: | Medicine/health sciences-related; Non-medicine/health sciences-related | Medicine/health sciences-related=1; non-related reference | Academic major |
| Q6 | Main financial support | 6、您最主要的经济来源是: | Parental support; part-time employment; student loan; scholarship; support from relatives/friends; other | Dummy variables; parental support reference | Main financial support |
| Q7 | Self-reported contraceptive familiarity | 7、您是否了解安全期、体外排精、工具及药物等避孕方法? | Very familiar; Generally familiar; Not very familiar; Do not understand at all | Primary binary outcome: higher familiarity=very/generally familiar; lower familiarity=other responses | Contraceptive familiarity; Higher self-reported contraceptive familiarity |
| Q8 | School-based education participation | 8、您是否参加过学校开展的生殖健康教育活动? | Participated; Did not participate; School did not offer activities | Descriptive variable; sensitivity-model dummies with did not participate as reference | School education participation |
| Q9 | Perceived need for reproductive health education | 9、您认为自己需要接受生殖健康教育吗? | Need; Indifferent; Do not need | Need=1; indifferent/do not need=0 | Perceived need for reproductive health education |
| Q10 | Perceived necessity of a school course | 10、你觉得学校有必要开展生殖健康方面的课程吗? | Necessary; Not necessary | Necessary=1; not necessary=0 | School course necessary |
| Q11 | Primary information source | 11、您现在所了解的有关生殖健康的知识，主要是通过以下哪个渠道? | School education; Internet; popular science books; family education; peers/friends; radio/television | Dummy variables; school education reference | Information source |
| Q12 | Preferred education topics | 12、您最想了解哪些方面的生殖健康知识? | Multiple response: healthy sexual behavior; STI/gynecological disease prevention; contraception; reproductive anatomy/function; sexual dysfunction; infertility treatment; sexual psychology; sexual ethics; sexual physiology/development; does not wish to learn any topic | Each option coded 1=selected, 0=not selected; no-topic response not used as a valid no-need outcome | Ten topic indicator variables |

*Note. The English item wording is provided in Supplementary File S1. Q7 measures self-reported familiarity, not objectively verified contraceptive knowledge.*

# Supplementary Table S2

Complete multivariable logistic regression coefficients for the three primary outcomes. All models include N=6,428 participants and use school education as the information-source reference category.

**Supplementary Table S2. Complete coefficients from primary multivariable logistic regression models for the three outcomes**

| **Characteristic** | **Familiarity B** | | **OR (95% CI)** | ***P*** | | **Education need B** | **OR (95% CI)** | ***P*** | | **Course necessary B** | **OR (95% CI)** | | ***P*** |
| --- | --- | --- | --- | --- | --- | --- | --- | --- | --- | --- | --- | --- | --- |
| Age, per 1-year increase | 0.072 | | 1.075 (1.056-1.094) | <0.001 | | 0.107 | 1.112 (1.094-1.131) | <0.001 | | 0.156 | 1.169 (1.146-1.193) | | <0.001 |
| Information source: Family education vs school education | -0.295 | | 0.744 (0.621-0.892) | 0.001 | | -0.044 | 0.957 (0.804-1.138) | 0.617 | | -0.445 | 0.641 (0.525-0.782) | | <0.001 |
| Information source: Internet vs school education | -0.051 | | 0.951 (0.811-1.114) | 0.530 | | 0.065 | 1.067 (0.919-1.239) | 0.396 | | 0.012 | 1.012 (0.846-1.211) | | 0.895 |
| Information source: Peers/friends vs school education | -0.309 | | 0.734 (0.608-0.885) | 0.001 | | -0.252 | 0.777 (0.648-0.932) | 0.006 | | -0.356 | 0.701 (0.570-0.861) | | <0.001 |
| Information source: Popular science books vs school education | 0.041 | | 1.042 (0.875-1.240) | 0.647 | | -0.197 | 0.821 (0.697-0.967) | 0.018 | | -0.271 | 0.763 (0.630-0.923) | | 0.005 |
| Information source: Radio/television vs school education | -0.311 | | 0.733 (0.582-0.922) | 0.008 | | -0.096 | 0.908 (0.726-1.136) | 0.400 | | -0.539 | 0.583 (0.456-0.745) | | <0.001 |
| Educational level: Junior college vs undergraduate | -0.230 | | 0.794 (0.703-0.898) | <0.001 | | 0.215 | 1.240 (1.101-1.398) | <0.001 | | -0.022 | 0.978 (0.857-1.117) | | 0.747 |
| Educational level: Master's degree or above vs undergraduate | -0.062 | | 0.940 (0.820-1.077) | 0.370 | | 0.149 | 1.161 (1.021-1.321) | 0.023 | | -0.062 | 0.940 (0.810-1.091) | | 0.413 |
| Rural vs urban household registration origin | -0.006 | | 0.994 (0.895-1.103) | 0.903 | | 0.100 | 1.105 (0.999-1.222) | 0.053 | | -0.060 | 0.941 (0.840-1.055) | | 0.298 |
| Only child: Yes vs no | 0.145 | | 1.156 (1.041-1.285) | 0.007 | | 0.113 | 1.120 (1.012-1.240) | 0.028 | | 0.181 | 1.198 (1.069-1.343) | | 0.002 |
| Medicine/health sciences-related vs non-related major | -0.345 | | 0.708 (0.637-0.788) | <0.001 | | -0.084 | 0.919 (0.829-1.019) | 0.108 | | -0.162 | 0.850 (0.757-0.955) | | 0.006 |
| Financial support: Other vs parental support | 0.191 | | 1.210 (0.765-1.914) | 0.414 | | 0.009 | 1.009 (0.668-1.525) | 0.965 | | 0.279 | 1.322 (0.739-2.366) | | 0.347 |
| Financial support: Part-time employment vs parental support | 0.026 | | 1.027 (0.886-1.189) | 0.726 | | -0.129 | 0.879 (0.764-1.010) | 0.069 | | -0.314 | 0.730 (0.624-0.855) | | <0.001 |
| Financial support: Scholarship vs parental support | -0.355 | | 0.701 (0.580-0.847) | <0.001 | | -0.288 | 0.750 (0.620-0.907) | 0.003 | | -0.580 | 0.560 (0.459-0.682) | | <0.001 |
| Financial support: Student loan vs parental support | -0.169 | | 0.844 (0.707-1.009) | 0.062 | | -0.201 | 0.818 (0.687-0.974) | 0.024 | | -0.301 | 0.740 (0.612-0.894) | | 0.002 |
| Financial support: Relatives/friends vs parental support | 0.126 | | 1.134 (0.916-1.403) | 0.248 | | -0.129 | 0.879 (0.720-1.073) | 0.204 | | -0.374 | 0.688 (0.551-0.858) | | <0.001 |
| **Model** | | **N** | | | **-2 Log likelihood** | | **Likelihood-ratio chi-square (df)** | | ***P*** | | | **Nagelkerke R2** | |
| Higher self-reported contraceptive familiarity | | 6,428 | | | 8141.499 | | 273.600 (16) | | <0.001 | | | 0.057 | |
| Perceived need for reproductive health education | | 6,428 | | | 8632.514 | | 277.540 (16) | | <0.001 | | | 0.056 | |
| School course considered necessary | | 6,428 | | | 7195.375 | | 487.586 (16) | | <0.001 | | | 0.105 | |

*Note. B indicates the logistic regression coefficient. ORs are adjusted odds ratios with 95% confidence intervals. All models adjust for age, educational level, household registration origin, only-child status, academic major, and main financial support. School-based education participation (Q8) was not included in primary models due to conceptual overlap with the exposure and potential role as a mediator.*

# Supplementary Table S3

Sensitivity analyses additionally adjusted for Q8 school-based reproductive health education participation. This variable was excluded from the primary models because it conceptually overlaps with school education as the exposure and may lie on the pathway between source and outcome.

**Supplementary Table S3. Sensitivity analyses additionally adjusted for school-based reproductive health education participation (Q8)**

| **Outcome** | **Characteristic** | **Primary model, aOR (95% CI); P** | **Q8-adjusted model, aOR (95% CI); *P*** |
| --- | --- | --- | --- |
| Higher self-reported contraceptive familiarity | Internet | 0.951 (0.811-1.114); 0.530 | 0.968 (0.825-1.136); 0.692 |
|  | Popular science books | 1.042 (0.875-1.240); 0.647 | 1.023 (0.857-1.220); 0.803 |
|  | Family education | 0.744 (0.621-0.892); 0.001 | 0.753 (0.628-0.904); 0.002 |
|  | Peers/friends | 0.734 (0.608-0.885); 0.001 | 0.734 (0.607-0.887); 0.001 |
|  | Radio/television | 0.733 (0.582-0.922); 0.008 | 0.758 (0.601-0.956); 0.019 |
|  | Q8: Participated vs did not participate | Not included | 1.320 (1.161-1.500); <0.001 |
|  | Q8: School did not offer activities vs did not participate | Not included | 0.630 (0.550-0.721); <0.001 |
| Perceived need for reproductive health education | Internet | 1.067 (0.919-1.239); 0.396 | 1.084 (0.932-1.260); 0.294 |
|  | Popular science books | 0.821 (0.697-0.967); 0.018 | 0.807 (0.684-0.951); 0.011 |
|  | Family education | 0.957 (0.804-1.138); 0.617 | 0.966 (0.811-1.151); 0.702 |
|  | Peers/friends | 0.777 (0.648-0.932); 0.006 | 0.776 (0.646-0.932); 0.007 |
|  | Radio/television | 0.908 (0.726-1.136); 0.400 | 0.938 (0.748-1.175); 0.575 |
|  | Q8: Participated vs did not participate | Not included | 1.372 (1.217-1.548); <0.001 |
|  | Q8: School did not offer activities vs did not participate | Not included | 0.767 (0.671-0.877); <0.001 |
| School course considered necessary | Internet | 1.012 (0.846-1.211); 0.895 | 1.038 (0.866-1.243); 0.689 |
|  | Popular science books | 0.763 (0.630-0.923); 0.005 | 0.747 (0.617-0.906); 0.003 |
|  | Family education | 0.641 (0.525-0.782); <0.001 | 0.652 (0.533-0.797); <0.001 |
|  | Peers/friends | 0.701 (0.570-0.861); <0.001 | 0.705 (0.573-0.868); <0.001 |
|  | Radio/television | 0.583 (0.456-0.745); <0.001 | 0.605 (0.472-0.774); <0.001 |
|  | Q8: Participated vs did not participate | Not included | 1.331 (1.157-1.531); <0.001 |
|  | Q8: School did not offer activities vs did not participate | Not included | 0.654 (0.567-0.755); <0.001 |

*Note. All estimates are adjusted odds ratios (95% confidence intervals); P values follow semicolons. School education is the information-source reference category. The primary-model covariates are retained in all sensitivity models. N=6,428 for every model. The Q8 reference category is did not participate. Nagelkerke R2 values for the Q8-adjusted models were 0.083, 0.073, and 0.126, respectively. These conditional estimates should be interpreted with caution, as the temporal and causal relationship between Q8 (past participation) and Q11 (current primary source) cannot be established in this cross-sectional design.*

# Supplementary Table S4

The approximate Brant-Wald test rejected the proportional-odds assumption (chi-square=128.708, df=32, *P*<0.001); therefore, ordinal estimates are presented as a diagnostic sensitivity analysis, whereas multinomial estimates provide category-specific contrasts.

**Supplementary Table S4. Ordinal and multinomial logistic regression sensitivity analyses for the four-level contraceptive familiarity outcome**

| **Outcome level** | **Internet** | **Popular science books** | **Family education** | **Peers/friends** | **Radio/television** |
| --- | --- | --- | --- | --- | --- |
| Ordinal logistic | 1.014 (0.890-1.156); 0.829 | 0.945 (0.820-1.090); 0.436 | 1.032 (0.885-1.204); 0.685 | 0.975 (0.829-1.146); 0.760 | 1.078 (0.882-1.318); 0.461 |
| Multinomial logistic |  |  |  |  |  |
| Not very familiar vs do not understand | 0.854 (0.656-1.110); 0.237 | 1.014 (0.758-1.358); 0.924 | 0.792 (0.592-1.061); 0.118 | 0.685 (0.508-0.923); 0.013 | 0.791 (0.552-1.133); 0.201 |
| Generally familiar vs do not understand | 0.788 (0.622-0.999); 0.049 | 1.059 (0.815-1.377); 0.667 | 0.572 (0.437-0.749); <0.001 | 0.500 (0.380-0.658); <0.001 | 0.533 (0.381-0.747); <0.001 |
| Very familiar vs do not understand | 0.963 (0.754-1.229); 0.763 | 1.018 (0.774-1.339); 0.897 | 0.750 (0.570-0.988); 0.040 | 0.720 (0.545-0.950); 0.020 | 0.779 (0.556-1.092); 0.148 |

*Note. School education is the reference category. All models adjust for age, educational level, household registration origin, only-child status, academic major, and main financial support. The approximate Brant-Wald test for the proportional-odds assumption was statistically significant (chi-square=128.708, df=32, P<0.001). Consequently, the ordinal logistic estimates should be interpreted cautiously; the multinomial logistic regression provides category-specific estimates without imposing proportional odds. The multinomial results indicate that the associations observed in the binary analysis were primarily driven by differences between the "generally familiar" and "do not understand" levels, with less pronounced differences at the "very familiar" threshold (see Discussion for interpretation). N=6,428.*

# Supplementary Table S5

# Response-consistency check for the preferred education-topics item. Of 1,760 participants selecting "does not wish to learn any topic," 1,688 (95.91%) also selected at least one specific topic. This response was retained only for transparent reporting and was not used to recode the separate education-need outcome or interpreted as a substantive topic preference.

**Supplementary Table S5. Cross-tabulation of the "Does not wish to learn any topic" response (Q12) and selection of at least one specific education topic**

| **Selection of specific topic(s)** | **Selected no-topic response** | **Did not select no-topic response** | **Total** |
| --- | --- | --- | --- |
| Selected at least one specific topic | 1,688 (95.91) | 4,498 (96.36) | 6,186 (96.24) |
| Selected no specific topic | 72 (4.09) | 170 (3.64) | 242 (3.76) |
| **Total** | **1,760 (100.00)** | **4,668 (100.00)** | **6,428 (100.00)** |

*Note. Data are n (% within the Q12 no-topic-response category). Of the 1,760 participants who selected the no-topic response, 1,688 (95.91%) also selected at least one specific topic. Therefore, the no-topic response is retained for descriptive transparency only and is not treated as a valid indicator of no education need, nor was it used to recode the separate education-need outcome (Q9).*

**Supplementary Figure S1. Comparison of primary (▲) and Q8-adjusted (●) models for associations between primary information source and three reproductive health outcomes.** Points denote adjusted odds ratios; horizontal bars indicate 95% confidence intervals. Models adjust for age, educational level, household registration origin, only-child status, academic major, and main financial support; the sensitivity model additionally adjusts for Q8. School education is the reference category; the horizontal axis is logarithmically scaled.

# Supplementary Figure S2. Participant flow diagram for the online cross-sectional survey of female college students in China. A total of 6,608 questionnaires were submitted; 180 were excluded based on predefined data-quality criteria (135 with logically inconsistent responses, 45 with completion time <120 seconds). The remaining 6,428 questionnaires (97.28%) were retained for analysis. The 97.28% value represents the proportion retained among submitted questionnaires and is not a survey response rate.

**Supplementary File S1: Bilingual Questionnaire Items(Chinese and English)**

Selected questionnaire items used in the present analysis. English translations are provided for international reporting. This file contains the original Chinese questionnaire items and their English translations for the variables used in the present analysis.

| **Item** | **OriginalChineseitem** | **Englishtranslation** | **Responseoptions** |
| --- | --- | --- | --- |
| Q1 | 1、(1)您的年龄:___周岁。 | What is your age (in completed years)? | Open numeric response |
| Q2 | 2、您现在就读的年级? | What is your current educational level? | 专科 / 本科 / 硕士及以上; Junior college / Undergraduate / Master's degree or above |
| Q3 | 3、您的原户籍所在地: | What is your original household registration location? | 城镇 / 农村; Urban / Rural |
| Q4 | 4、您是独生子女吗? | Are you an only child? | 是 / 不是; Yes / No |
| Q5 | 5、您所学专业是: | What is your academic major? | 医学卫生相关专业 / 非医学卫生相关专业; Medicine/health sciences-related / Non-medicine/health sciences-related |
| Q6 | 6、您最主要的经济来源是: | What is your main financial support source? | 父母提供 / 亲戚朋友资助 / 兼职收入 / 助学贷款 / 奖学金 / 其它; Parental support / relatives or friends / part-time employment / student loan / scholarship / other |
| Q7 | 7、您是否了解安全期、体外排精、工具及药物等避孕方法? | How familiar are you with contraceptive methods, such as the safe period, withdrawal, barrier methods, and medications? | 非常了解 / 一般了解 / 不大了解 / 一点不懂; Very familiar / Generally familiar / Not very familiar / Do not understand at all |
| Q8 | 8、您是否参加过学校开展的生殖健康教育活动? | Have you participated in reproductive health education activities organized by your school? | 参加过 / 没有参加过 / 学校没有开展过; Participated / Did not participate / School did not offer activities |
| Q9 | 9、您认为自己需要接受生殖健康教育吗? | Do you think you need reproductive health education? | 需要 / 无所谓 / 不需要; Need / Indifferent / Do not need |
| Q10 | 10、你觉得学校有必要开展生殖健康方面的课程吗? | Do you think it is necessary for schools to offer reproductive health courses? | 有必要 / 没有必要; Necessary / Not necessary |
| Q11 | 11、您现在所了解的有关生殖健康的知识，主要是通过以下哪个渠道? | What is the main channel through which you currently obtain reproductive health information? | 科普读物 / 网络 / 同学及朋友 / 广播电视 / 家庭教育 / 学校教育; Popular science books / Internet / peers or friends / radio or television / family education / school education |
| Q12 | 12、您最想了解哪些方面的生殖健康知识? | Which aspects of reproductive health knowledge would you most like to learn about? (Multiple responses allowed) | 健康的性行为; 性病及妇科病预防知识; 避孕知识; 男女生殖器解剖及基本功能; 性功能障碍; 不孕不育的治疗; 性心理知识; 性道德伦理; 性生理及发育知识; 不想了解任何知识. / Healthy sexual behavior; prevention of sexually transmitted infections and gynecological diseases; contraception; reproductive anatomy and basic function; sexual dysfunction; infertility treatment; sexual psychology; sexual ethics; sexual physiology and development; does not wish to learn any topic. |

Translation note. Q7 is reported as self-reported contraceptive familiarity because the question asks participants to rate their familiarity; it does not objectively test contraceptive knowledge. Q12 contains a response-consistency issue documented in Supplementary Table S4.
